# Best clinical practice guidance for clinicians dealing with children presenting with molar-incisor-hypomineralisation (MIH): an updated European Academy of Paediatric Dentistry policy document

**DOI:** 10.1007/s40368-021-00668-5

**Published:** 2021-10-20

**Authors:** N. A. Lygidakis, E. Garot, C. Somani, G. D. Taylor, P. Rouas, F. S. L. Wong

**Affiliations:** 1 Private Paediatric Dental Clinic, 2 Papadiamantopoulou Street, 11528 Athens, Greece; 2 Univ. de Bordeaux, UFR des Sciences Odontologiques, Bordeaux, France; 3 CHU de Bordeaux, Pôle médecine et chirurgie bucco-dentaire, Pellegrin, Bordeaux, France; 4 Univ. de Bordeaux, PACEA, UMR 5199, Pessac, France; 5 Paediatric Dentistry, Institute of Dentistry, Barts and The London School of Medicine and Dentistry, Queen Mary University of London, London, UK; 6 School of Dental Sciences, Faculty of Medical Sciences, Newcastle University, Newcastle upon Tyne, UK

**Keywords:** Molar incisor hypomineralisation, MIH, Clinical practice, Treatment, Aetiology, Policy document, EAPD, Guidelines

## Abstract

**Aim:**

To update the existing European Academy of Paediatric Dentistry (EAPD) 2010 policy document on the ‘Best Clinical Practice guidance for clinicians dealing with children presenting with Molar-Incisor-Hypomineralisation (MIH).’

**Methods:**

Experts, assigned the EAPD, worked on two different topics: (A) Aetiological factors involved in MIH, and (B) Treatment options for the clinical management of MIH. The group prepared two detailed systematic reviews of the existing literature relevant to the topics and following a consensus process produced the updated EAPD policy document on the ‘Best Clinical Practice guidance for clinicians dealing with children presenting with molar-incisor-hypomineralisation (MIH).’ The GRADE system was used to assess the quality of evidence regarding aetiology and treatment which was judged as HIGH, MODERATE, LOW or VERY LOW, while the GRADE criteria were used to indicate the strength of recommendation regarding treatment options as STRONG or WEAK/CONDITIONAL.

**Results:**

(A) Regarding aetiology, it is confirmed that MIH has a multifactorial aetiology with the duration, strength and timing of occurrence of the aetiological factors being responsible for the variable clinical characteristics of the defect. Perinatal hypoxia, prematurity and other hypoxia related perinatal problems, including caesarean section, appear to increase the risk of having MIH, while certain infant and childhood illnesses are also linked with MIH. In addition, genetic predisposition and the role of epigenetic influences are becoming clearer following twin studies and genome and single-nucleotide polymorphisms analyses in patients and families. Missing genetic information might be the final key to truly understand MIH aetiology. (B) Regarding treatment options, composite restorations, preformed metal crowns and laboratory indirect restorations provide high success rates for the posterior teeth in appropriate cases, while scheduled extractions provide an established alternative option in severe cases. There is great need for further clinical and laboratory studies evaluating new materials and non-invasive/micro-invasive techniques for anterior teeth, especially when aesthetic and oral health related quality of life (OHRQoL) issues are concerned.

**Conclusions:**

MIH has been studied more extensively in the last decade. Its aetiology follows the multifactorial model, involving systemic medical and genetic factors. Further focused laboratory research and prospective clinical studies are needed to elucidate any additional factors and refine the model. Successful preventive and treatment options have been studied and established. The appropriate choice depends on the severity of the defects and the age of the patient. EAPD encourages the use of all available treatment options, whilst in severe cases, scheduled extractions should be considered.

**Supplementary Information:**

The online version contains supplementary material available at 10.1007/s40368-021-00668-5.

## Background

The European Academy of Paediatric Dentistry (EAPD) was the first international scientific organisation that extensively studied molar-incisor- hypomineralisation (MIH) and developed a policy document and relevant publications. The first policy document was published in 2010 being produced after an Interim Seminar and Workshop organised by the EAPD in Helsinki in May 2009 (Lygidakis et al. 2010). At that time, a comprehensive search of the literature was undertaken and presented by the invited speakers. This revealed a limited number of high-quality research studies on the topic. Due to the small number, and their shortcomings, it was evident that a ‘Guidelines Diagram’ according to the SIGN Methodology, popular at the time, was impossible to be made. However, it was agreed by all of the workshop speakers and experts that there was a great need for a guide to support clinicians encountering children with MIH. This led to the ‘Best Clinical Practice Guidance’ being developed and was based on a combination of available current evidence and consensus expert opinion from those attending the workshop. The document became extremely popular and is still used internationally by clinicians dealing with MIH.

Ten years later, in 2019, the EAPD assigned two of the previous experts to lead a study group to update the existing ‘Best Clinical Practice Guidance’. The study group, comprising six experts, worked intensively for eight months prior to the 12th EAPD Interim Seminar in Oslo, on two different topics: (A) aetiological factors involved in MIH, and (B) treatment options for the clinical management of MIH. The group prepared two detailed systematic reviews and meta-analyses, where appropriate, of the existing literature relevant to the topics (Garot et al. 2021; Somani et al. 2021). Thereafter, and following a consensus process, the updated EAPD policy document on the ‘Best Clinical Practice guidance for clinicians treating children with molar incisor hypomineralisation (MIH)’ was produced and presented at the 12th EAPD Interim Seminar in Oslo. The document was assessed by the Clinical Affairs Committee of EAPD for further comments and finally published in the European Archives of Paediatric Dentistry.

## Methods

Instead of the SIGN methodology, that was used in 2009, the present updated ‘MIH best clinical practice guidance’ employed the GRADE system (Guyatt et al. 2008; Ryan and Hill 2016) to assess the quality of existing evidence for both the aetiology and the treatment options applied in MIH. The quality of evidence was judged as HIGH, MODERATE, LOW or VERY LOW, based on assessment of eight criteria which may have an impact to the confidence in the results. These criteria are risk of bias, indirectness, inconsistency, imprecision, publication bias, large magnitude of effect, dose response and the effect of all plausible confounding factors for reducing the effect or suggest a spurious effect. Following the quality assessment, the same approach was used to indicate the strength of recommendation for each treatment option available, as STRONG or WEAK/CONDITIONAL. The interpretation of the gradings for quality of evidence and strength of recommendation are shown in Tables 1 and 2. Details of all studies evaluated and the methodology implemented, are included in the two relevant systematic reviews prepared by the study group (Garot et al. 2021; Somani et al. 2021).

**Table 1:** GRADE ratings and their interpretation

Grades of evidence quality	Interpretation
High	We are very confident that the true effect lies close to that of the estimate of the effect
Moderate	We are moderately confident in the effect estimate: the true effect is likely to be close to the estimate of the effect, but there is a possibility that it is substantially different
Low	Our confidence in the effect estimate is limited: the true effect may be substantially different from the estimate of the effect
Very low	We have very little confidence in the effect estimate: the true effect is likely to be substantially different from the estimate of effect

Table from the GRADE Handbook, available at: http://gdt.guidelinedevelopment.org/app/handbook/handbook.html#h.9rdbelsnu4iy

**Table 2:** Strengths of recommendation for patients and clinicians

	Strong recommendation	Weak/Conditional recommendation
For patients	Most people would want the recommended course of action and only a small proportion would not	Most people would want the recommended course of action, but many would not
For clinicians	Most patients should receive the recommended course of action	Different choices will be appropriate for different patients and each patient should be advised for a management decision consistent with her/his values and preferences

Adapted from: Guyatt et al. GRADE Working Group. Rating quality of evidence and strength of recommendations: going from evidence to recommendations. BMJ.2008; 336:1049–51

## Terminology and diagnosis of MIH

### Terminology

Regarding terminology, EAPD at present reinforces the use of the term MIH, as it has been established previously by the EAPD criteria (Weerheijm et al. 2003; Lygidakis et al. 2010) and it is well known and adopted globally. However, as it was proposed in the previous policy document, there is a risk that the current definition of MIH could be misleading and may result in an under-estimation of the defect. Demarcated opacities, similar to MIH, have been observed in the tips of permanent canine cusps, second permanent molars and the premolars. Future workshops should focus their efforts to formulate a more inclusive term. In addition, the term HSPM (hypo-mineralised second primary molars) has now been clearly established indicating demarcated opacities of the same type as MIH on second primary molars (Elfrink et al. 2012). Despite the limitations of the few published studies, it has been clearly shown that the presence of HSPM is predictive for MIH, with higher MIH prevalence in the presence of mild HSPM (Garot et al. 2018).

### Diagnostic criteria

EAPD reinforces the use of the specific clinical signs and symptoms for the diagnosis of MIH, as previously described in the earlier EAPD publications (Weerheijm et al. 2003; Lygidakis et al. 2010). The use of intraoral X-Rays may add to the diagnosis (Aps et al. 2020). These criteria are shown in Table 3.

**Table 3:** EAPD Diagnostic criteria of MIH (adopted from Weerheijm et al. 2003; Lygidakis et al. 2010)

Diagnostic feature	Description of the defect
Teeth involved	One to all four permanent first molars (FPM) with enamel hypomineralisation Simultaneously, the permanent incisors can be affected At least one FPM has to be affected for a diagnosis of MIH The more affected the molars, the more incisors involved and the more severe the defects The defects may also be seen at the second primary molars, premolars, second permanent molars and the tip of the canines
Demarcated opacities	Clearly demarcated opacities presenting with an alteration in the translucency of the enamel Variability in colour, size and shape White, creamy or yellow to brownish colour Only defects greater than 1 mm should be considered
Post-eruptive enamel breakdown	Severely affected enamel breaks down following tooth eruption, due to masticatory forces Loss of the initially formed surface and variable degree of porosity of the remaining hypomineralised areas The loss is often associated with a pre-existing demarcated opacity Areas of exposed dentine and subsequent caries development
Sensitivity	Affected teeth frequently reveal sensitivity, ranging from mild response to external stimuli to spontaneous hypersensitivity MIH molars may be difficult to anesthetize
Atypical restorations	The size and shape of restorations are not conforming to the typical caries picture In molars the restorations are extended to the buccal or palatal/lingual smooth surface An opacity can be frequently noticed at the margins of the restorations First permanent molars and incisors with restorations having similar extensions as MIH opacities are recommended to be judged as that
Extraction of molars due to MIH	Extracted teeth can be defined as having MIH when there are: - Relevant notes in the records - Demarcated opacities or atypical restorations on the other first molars - Typical demarcated opacities in the incisors

### Severity of the defects

EAPD reinforces the baseline classification of the defects as mild or severe (Table 4), as they have been detailed previously, to clarify the diagnosis and formulate an appropriate treatment plan (Lygidakis et al. 2010).

**Table 4:** Description of severity level according to the EAPD criteria (adopted from Jälevik 2010; Lygidakis et al. 2010)

Severity level	Signs and symptoms
Mild	Demarcated enamel opacities without enamel breakdown Induced sensitivity to external stimuli e.g., air/water but not brushing Mild aesthetic concerns on discolouration of the incisors
Severe	Demarcated enamel opacities with breakdown and caries Spontaneous and persistent hypersensitivity affecting function e.g., brushing, mastication Strong aesthetic concerns that may have socio-psychological impact

### Recording MIH for epidemiological studies

**Fig. 1:**
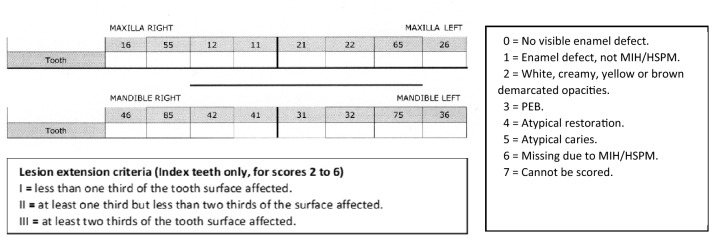
MIH/HSPM clinical data short recording sheet and scoring details. Only teeth involved in MIH/HSPM and the relevant MIH characteristics are included (Ghanim et al. 2015)

**Fig. 2:**
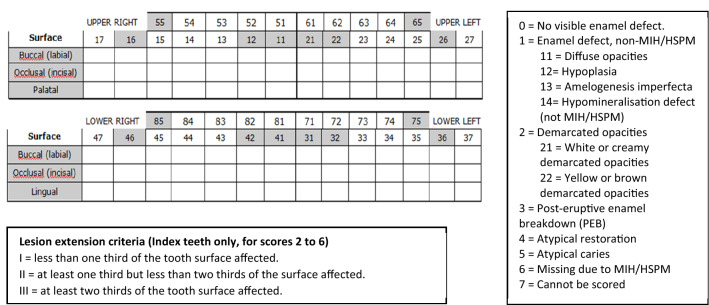
MIH/HSPM clinical data long recording sheet and scoring details. Teeth involved in MIH/HSPM are highlighted grey. MIH and mDDE characteristics in all existing teeth are included (Ghanim et al. 2015)

Regarding epidemiological studies, the approach presented by Ghanim et al. (2015) seems to be the most appropriate at present, as it combines the well-established elements of the EAPD criteria and the modified index of developmental defects of enamel (mDDE) (Clarkson and O’Mullane 1989). This approach grades the clinical status of MIH and its extent on the involved tooth surface as well as other enamel defects and additionally includes any existence of HSPM in the same child (Ghanim et al. 2015). The basic features are shown in Figs. 1 and 2. To take into account the varied needs and objectives of epidemiological studies, two forms of the examination chart have been proposed, a short form for simple screening surveys using only the EAPD criteria and a long form for more detailed community-based or clinic-based studies that use both EAPD and mDDE criteria (Ghanim et al. 2015, 2017). The short data form is designed to grade only index teeth which have been mentioned in the definition of MIH and HSPM, namely first permanent molars, permanent incisors, and second primary molars. The long data form is formulated to diagnose all teeth at surface level available at the time of the dental examination in addition to MIH/HSPM-specific index teeth.

Recently, one further index has been developed that is useful for determining mainly the treatment needs of children with MIH (Steffen et al. 2017). The index is defined as MIH Treatment Need Index (MIH-TNI) and has been designed for identifying patients with MIH, providing information about the severity, and describing their subsequent treatment needs.

## Prevalence data of MIH

The first epidemiological data, from national studies carried out in European countries, reported prevalence varying from 3.6 to 25% (Weerheijm and Mejàre 2003). Subsequent reviews of the literature showed an even wider range in the worldwide MIH prevalence (2.9–44%) (Jälevik 2010; Elfrink et al. 2015). Comparing the results of different studies in the past was difficult because of the use of different indices, diagnostic criteria, registration methods and age groups. In the last decade, the number of studies evaluating the prevalence of MIH at national or regional level has significantly increased. In addition, most studies are now routinely using the standardised EAPD diagnostic and epidemiological criteria for MIH (Elfrink et al. 2015; Ghanim et al. 2017). Two recent systematic reviews and meta-analyses revealed similar global mean prevalence; the first one 14.2% (8.1–21.1%) (Zhao et al. 2018; Dave and Taylor 2018) and the second 12.9% (11.7–14.3%) (Schwendicke et al. 2018). Both reviews established significant prevalence differences between super-regions, regions and countries, while certain countries, mainly in regions with limited access to dental care, shoulder the majority of new cases of MIH.

**Fig. 3:**
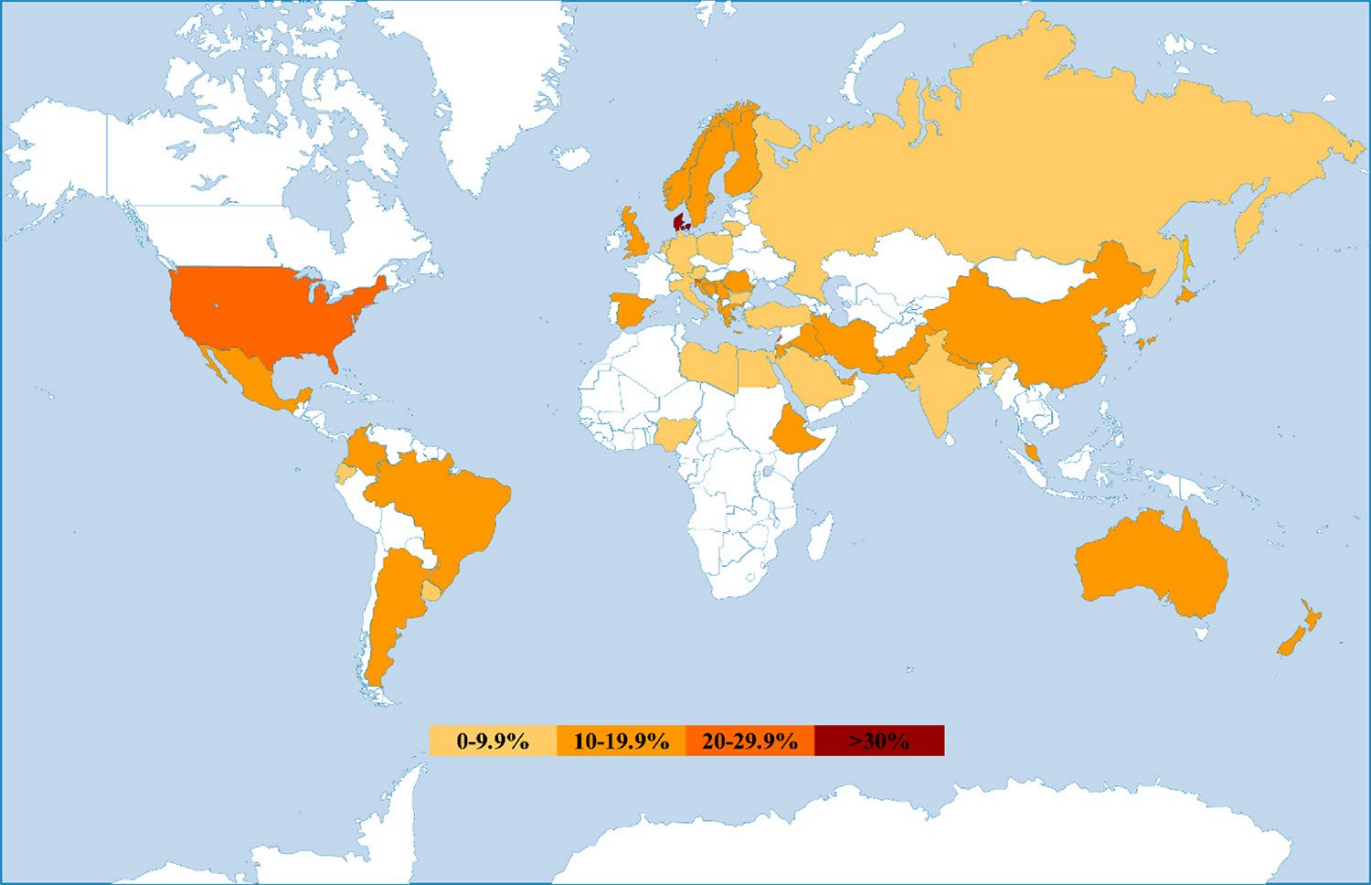
World map of MIH prevalence at present

To show the global MIH prevalence at present, the weighted mean for each country/region was calculated using existing data and is shown using a colour gradient on a world map (Fig. 3). In the most recent prevalence systematic review (Schwendicke et al. 2018), only the studies from 2000 to May 2017 were included. In preparation of this policy document, it was noted that more prevalence studies have been published between May 2017 and September 2020. It was agreed by all authors of this policy document to include these additional prevalence figures in our world prevalence map.

Evaluating all existing prevalence data, it appears that MIH is still not confidently diagnosed by general practitioners, in contrast to paediatric dentists who are more familiar with the condition. Patients sometimes present with severe destruction in their first permanent molars, atypical restorations, or even with extracted molars. Such cases, together with the absence of opacities, could be misdiagnosed as caries. Therefore, the prevalence of MIH could be underestimated, unless clinicians with more experience and training in observing MIH diagnosis are involved in national epidemiological studies (Zhao et al. 2018).

It is worth noting that the current mean prevalence figures of MIH are very close to that highlighted in the first reported prevalence study in 1987, although the established MIH EAPD diagnostic criteria did not exist at that time; their findings estimated a prevalence 13–16% following examination of 2226 patients born between 1966 and 1974 in Sweden (Koch et al. 1987). This comparison, added to the absence of any relevant scientific evidence, indicates that over time the prevalence of MIH may not have increased, despite opposing suggestions.

## Aetiology of MIH

### General considerations

In the 2010 ‘policy document’, it was stated that ‘It is likely that MIH is not caused by one specific factor. Several harmful agents/conditions may act together and increase the risk of MIH occurring additively or even synergistically.’ (Lygidakis et al. 2010). Since 2010, data from more aetiological studies support and confirm this statement (Table 5). The evidence now re-affirms that in MIH, certain systemic and genetic factors act synergistically to produce enamel hypomineralisation. In addition, the duration, strength and timing of these factors may be responsible for the varied clinical characteristics of the defect.

**Table 5:** GRADE quality of evidence of included studies related to aetiology

Aetiological factor (in alphabetical order)	No of studies	No of participants	No of participants with the factor	Quality of evidence
Allergies	6	2432	518	Low
Antibiotics	19	11,703^a^	2330	Low
Asthma	14	8104^a^	1227	Low
Asthma drug	5	3669	981	Low
Breast feeding > 12 months	8	8189	972	Low
Breast feeding > 6 months	7	4810	928	Low
Bronchitis	12	9473^a^	1270	Low
Caesarean	17	10,886^a^	2336	Low
Celiac disease	1	80	40	Very low
Chicken pox	14	9845	1437	Very low
Diarrhoea	7	6893	908	Low
Eclampsia	4	5409	592	Low
Epigenetic (monozygotic twins)	1	334	188	High
Fever	20	14,128^a^	2408	Low
Fluoride	1	3233	2507	Low
Gastric disorders	8	6266	647	Low
Genetic (SNP association)	4	1456	ND	High
Gestational diabetes	4	1554	406	Very low
Gestational hypertension	9	7611	1009	Low
Hypoxia at birth	16	9867^a^	1859	Low
Incubator	5	5628	702	Moderate
Jaundice	4	1220	220	Very low
Kidney diseases	7	3758	754	Low
Low birth weight	11	10,150^a^	1779	Low
Malnutrition	4	1464	342	Very low
Maternal diseases	14	15,312^a^	1853	Low
Maternal fever	4	4921	536	Low
Maternal smoking	6	4227	1278	Low
Maternal Urinary disease	4	5410	592	Low
Measles	3	4139	348	Low
Medication during pregnancy	8	3879	722	Moderate
Otitis	17	9421^a^	1417	Low
Pneumonia	12	10,021^a^	1581	Low
Pre-eclampsia	7	7517	1042	Low
Prematurity	19	12,307^a^	2405	Low
Rhinitis	5	3281	600	Low
Rubeola	2	5338	516	Low
Sinusitis	3	1401	311	Low
Throat infections	3	2403	405	Low
Tonsillitis	4	1290	261	Very low
Urinary tract infection	11	8675^a^	1015	Low
Vitamin D deficiency	1	1840	ND	High

^a^Studies with large number of examined children where (regardless the low quality of evidence resulting from the retrospective methodology) the meta-analysis revealed odds ratios indicating increased risk of having MIH (Garot et al. 2021).

More than 30 systemic aetiological hypotheses have been identified over the last 10 years; some are well established, others are more contemporary. The different aetiological hypotheses can be linked to the pre-, peri- and post-natal periods, as alterations in the function of the ameloblasts during the maturation phase may occur between the end of pregnancy and the age of 4 years (Alaluusua 2010).

### Genetics/Epigenetics

There has been an increase in research focusing on the genetics associated with the aetiology of MIH. The levels of MIH-affected teeth observed between monozygotic and dizygotic twins in clinical studies (Teixeira et al. 2018), infer the relative importance of genetics. It is worth noting, however, that such studies have certain methodology limitations and twins are more likely to encounter similar proposed aetiological factors in the peri- and post-natal periods when compared to non-twin births (Lygidakis et al. 2010).

Recent studies evaluated single-nucleotide polymorphisms (SNP) in a group of individuals with and without MIH (Jeremias et al. 2013a, b, 2016). The SNP corresponds to the variation (polymorphism) of a single base pair in the genome, and could be the basis of our species' susceptibility to certain diseases. The genetic associations between SNP rs3790506 (TUFT1) and SNP rs946252 (AMELX) and MIH were investigated but no association between these SNPs and MIH was demonstrated (Jeremias et al. 2013a, b). However, in a later study, the same authors established a link between the rs5979395 SNP of the AMELX gene (Xq22) and MIH (OR 11.7; *P* = 0.006) with 97% of these participants with MIH carrying the rs5979395*G allele (Jeremias et al. 2016). Other authors have identified the rs13058467 locus, located near the SCUBE1 gene on chromosome 22 (p<3.72E7), as a possible locus related to MIH (Kühnisch et al. 2014). The SCUBE1 gene plays a role in the development of the craniofacial region, and in a mouse model, it was found to be localised to the dental papilla of both incisor and molar teeth (Xavier et al. 2009). A genetic predisposition to MIH in conjunction with one or several other aetiological factors has been proposed, as some authors identified certain variants in amelogenesis-related genes ENAM, AMELX or MMP20 (Jeremias et al. 2013a, b, 2016; Kühnisch et al. 2014; Hočevar et al. 2020; Pang et al. 2020) or immune response-related genes (Bussaneli et al. 2019) in children with MIH. More recently, epigenetic influences of certain environmental factors have also been established (Teixeira et al. 2018; Vieira 2019; Vieira and Manton 2019). Epigenetics describes the way in which gene–environment and gene–gene interactions cause the expression of a phenotype. In other words, it is possible that epigenetics regulates the different systemic factors that influence the function of the enamel proteins involved in MIH (Kühnisch et al. 2014). In a recent study, it has been reported that individual variations in different genes have an additive effect on the development of MIH, which most likely occurs under the influence of specific environmental/systemic factors (Hočevar et al. 2020).

All the above suggest that MIH follows a multifactorial model with genetic and/or epigenetic components becoming more prominent in the more recently established evidence (Pang et al. 2020; Bussaneli et al. 2021).

### Systemic and medical aetiological factors

- Prenatal period

In a recent systematic review, no specific illness during the last trimester of pregnancy was associated with MIH (Garot et al. 2021). Additionally, there is no convincing evidence of an association between drugs taken during pregnancy, maternal smoking or maternal alcohol intake and MIH (Fatturi et al. 2019; Garot et al. 2021). It has been reported that certain medical problems are more frequently present in mothers of children with MIH (Whatling and Fearne 2008; Sönmez et al. 2013; Koruyucu et al. 2018; Fatturi et al. 2019; Mejia et al. 2019). However, what constituted as maternal illnesses differed substantially among the studies and most were determined retrospectively from interviewing the mother, thus introducing a recall bias.

b. Perinatal period

In the perinatal period, different parameters, such as hypoxia, premature birth, low birth weight, birth complications and caesarean section, presenting alone or in combination, appear to be associated with the presence of MIH (Garot et al. 2021). This recent systematic review and meta-analysis demonstrated that hypoxia at birth, reported in 16 studies (*n* = 9867 participants), substantially increased the possibility of having MIH (OR 2.76 [2.09, 3.64]; *P* < 0.0001) (Garot et al. 2021). Hypoxia may be associated with birth-related medical problems noted above, or in addition to prolonged delivery (Ananth and Chauhan 2012; Esteves-Pereira et al. 2021).

When analysed in isolation, prematurity was significantly associated with MIH (OR 1.45; 95% CI 1.24–1.70; *P* = 0.0002) (Garot et al. 2021). This finding is in contrast with that reported in a previous systematic review and meta-analysis (Fatturi et al. 2019). A similar conclusion was observed in relation to the positive effect of caesarean section (Garot et al. 2021), as it appeared not to be associated with MIH in the previous systematic review (Fatturi et al. 2019). The most recent review included 17 studies (*n* = 10,886 participants) and indicated that caesarean section increased the possibility of having MIH (OR 1.45; 95% CI 1.09–1.93; *P* = 0.01) (Garot et al. 2021).

These conflicting results could be explained by the number of included studies in the two reviews. Garot et al. (2021) included and evaluated almost twice as many studies to that by Fatturi et al. (2019), and therefore provides a stronger argument for known peri-natal systemic aetiological factors in the development of MIH (Table 5).

c. Postnatal period

During the post-natal period, many factors may intervene in the life of the child from birth to 4 years, the suggested critical period for MIH development (Lygidakis et al. 2008b; Whatling and Fearne 2008). The hypothetical aetiological factors include effects by environmental pollutants, childhood diseases and medication.

Previous studies have suggested that environmental pollutants found in breast milk might be associated with MIH (Alaluusua et al. 1996). However, none of the studies included in the most recent meta-analysis found a link between the duration of breastfeeding and the occurrence of MIH (Garot et al. 2021). This was a similar finding to that by Fatturi et al. (2019) who reported there was no link between breast-feeding and MIH.

With regard to the use of medication, only antibiotics have been linked to MIH (Fatturi et al. 2019; Garot et al. 2021). Childhood illnesses, such as measles, urinary tract infection, bronchitis, otitis, gastric disorders, fever, kidney diseases, pneumonia and asthma, were also found to be associated with MIH (Garot et al. 2021). It is known that antibiotics are prescribed to manage many of these conditions. Therefore, it is likely that the presence of a particular disease, rather than the antibiotics prescribed to manage it, is associated with MIH. In addition, no association between MIH and rubeola, sinusitis, jaundice, rhinitis, malnutrition, throat infections, allergies and diarrhoea was found in the most recent systematic review (Garot et al. 2021).

Recording the history of childhood diseases across most studies frequently relied on the recall of the parents, introducing a recall bias. Nevertheless, accepting this as a limitation of the current evidence, there appears to be a clear association between certain systemic factors and MIH in the post-natal period.

### Comments arising from the evaluation of the existing aetiology literature

#### Is there a link between the duration, strength and timing of aetiological factors action and the development of MIH?

In the previous ‘EAPD Policy Document’ of 2010, the following comment was made by the working group; ‘Is the aetiology of MIH likely to be a question of dosage or duration of the effect during a critical time in tooth development?’ (Lygidakis et al. 2010).

Recent evidence seems to support this statement. Given the asymmetrical nature of the hypomineralised defect in terms of location and severity, the hypothesis that short events result in less severe defects is supported by the studies of Sidaly et al. (2015, 2016). These studies have shown that a short episode of induced severe hypoxia (less than 5 min) in adult mice can cause a single confined area of enamel hypomineralisation on their incisors, with the position and character of the defect being related to the functional stage at which the ameloblasts are affected (Sidaly et al. 2016). In contrast, large-scale case–control studies have shown that the number of the affected teeth was greater when more aetiological factors were involved for longer periods (Lygidakis et al. 2008a). Similarly, in the prenatal period, repeated episodes of maternal high fever during pregnancy result in children having 2.54 times greater chance of developing severe MIH (Dantas-Neta et al. 2016).

In addition to systemic aetiological factors, genes and epigenetic actions appear to also mediate environmental influences on the gene expression. This might support the view that MIH occurs as a result of certain life events, while the mechanism linking systemic and genetic factors might explain the different severities of MIH in teeth forming at the same time although in different locations (Vieira 2019; Vieira and Manton 2019).

#### Are archaeological findings helpful in studying the aetiology of MIH?

The increasing number of MIH cases in archaeological skeletal remains in France and England may help to lower the significance of some reported aetiological factors, such as Bisphenol A and other endocrine disruptors, antibiotics, dioxins, and other pollutants, as these were not present in the medieval time (Ogden et al. 2008; Curzon et al. 2015; Garot et al. 2017, 2019). Some of these studies, besides clinical observations, undertook laboratory investigations to confirm the observational findings (Garot et al. 2017). There are, however, some opposing clinical observations (Kühnisch 2017), reporting a low prevalence of MIH in adults’ archaeological case series from Germany. Despite this inconsistency, this line of thinking should be further investigated, as it might easily help to exclude contemporary living conditions from the aetiology of MIH.

#### Are the current studies investigating systemic aetiological factors robust enough to draw definitive results and answers regarding MIH aetiology?

As previously stated, ‘The problem of most clinical studies relating MIH or MIH like lesions with medical conditions/problems, so far, is that they are retrospective. The information has been obtained by questionnaires or an interview, which rely on individual memory and can lead to inaccuracies’ (Alaluusua 2010). Unfortunately, a similar situation exists currently, as most studies are retrospective, relying on parent interviews to recall information (Garot et al. 2021). Such studies results are biased and, regardless of which statistical approach is undertaken, are usually compromised. Although the planning of prospective studies is indeed very difficult for such a long period of follow-up (from pregnancy up to until the age of 8 years), their future planning appears a necessity to further clarify the aetiology of MIH. The publication of more retrospective aetiological studies would not add anything new to the existing evidence base.

### GRADE rating for the quality of evidence of the aetiology studies in MIH

The GRADE system was used to assess the quality of studies on the aetiology of MIH which was judged as HIGH, MODERATE, LOW or VERY LOW (Table 5) based on the assessment of eight criteria which can influence the confidence of the results (Guyatt et al. 2008). Details of the implementation of all criteria regarding the included studies are shown in the supplement as Appendix 1.

## Treatment approaches for MIH teeth

- **Treatment of posterior teeth**

Appropriate treatment decisions for posterior teeth should take into consideration several factors (Table 6), arising from increased clinical knowledge and relevant clinical and laboratory studies.

**Table 6:** Factors to be considered for appropriate treatment planning for posterior teeth

At patient level	At oral level	At tooth level
Age of patient	Number of affected teeth	Size of defect
Medical history	DMFT	Location of defect
Ability to cooperate	Developmental stage	Number of surfaces involved
Presence/absence of symptoms	Occlusion	Presence/absence of post-eruptive breakdown
Access to general dental care	Presence/absence of crowding	Presence/absence of atypical or typical carious lesions and extent
Access to specialist care (paediatric dental/orthodontic)	Presence of third permanent molars	Pulpal involvement
	Hypodontia	History of dental abscess/facial cellulitis
	Need for future orthodontic treatment	

*Preventive approach* Early identification of MIH-affected teeth is key to the management of affected molars. As these teeth are at higher risk of caries (Jeremias et al. 2013a, b; Bullio Fragelli et al. 2015), early prevention is very important. Due to the presence of hypersensitivity, children may avoid oral hygiene procedures. Therefore, enhanced oral hygiene instruction and dietary advice should be provided and reinforced regularly to both children and their carers. Placement of topical fluoride varnish is also advised, primarily for preventing caries in permanent teeth (Marinho et al. 2013; Toumba et al. 2019). However a longitudinal study found that despite placement of fluoride varnish on MIH-affected molars, an increased risk of caries and post-eruptive breakdown was still observed (Bullio Fragelli et al. 2015). Combining topical fluoride varnish placement with frequent recall intervals of 3–6 months and enhanced preventive measures allow the practitioner to closely monitor the affected teeth for breakdown. Silver diamine fluoride has been used successfully in primary teeth to arrest caries (Seifo et al. 2019). However, there are currently no published clinical studies documenting its use on MIH-affected teeth. For fully erupted molars, resin-based fissure sealants should be considered the first line approach in preventing both dental caries and post-eruptive breakdown (Lygidakis et al. 2009; Fragelli et al. 2017). The use of an adhesive during fissure sealant placement is advised as it has been clearly shown to increase the retention rate (Lygidakis et al. 2009).

*Atraumatic restorations* Where a child lacks co-operation for invasive treatment requiring local anaesthetic, or is unable to access routine dental care, a glass ionomer cement (GIC) restoration can be placed in the interim, until a suitable restoration can be placed, or before an age-scheduled extraction, to protect the tooth from post-eruptive breakdown and hypersensitivity. As a hydrophilic material having the added benefit of fluoride release, GIC can be used where ideal moisture control is not possible. The inferior mechanical properties of GIC, however, preclude their use in stress-bearing areas. Non-invasive techniques have shown variable/poor success rates (Fragelli et al. 2015; Linner et al. 2020) but the atraumatic restorative technique has shown some promising short-term results using a glass-ionomer hybrid restoration (Grossi et al. 2018), or a high-viscosity GIC (Durmus et al. 2021). These studies have however methodological flaws and further high-quality research is needed to understand the true effectiveness of the atraumatic restorative technique, and materials used, in MIH.

*Considerations affecting treatment in severe cases* In severe cases where breakdown has already occurred or a cavity is present due to caries, a number of treatment options are available. Ultimately, when faced with this clinical scenario, a decision needs to be made as to whether to restore or extract these teeth. Such a decision needs to take all the factors noted in Table 6 into consideration. A recent study highlighted how complex these decisions are with substantial differences noted between and within a group of non-specialists and specialists in paediatric dentistry (Taylor et al. 2019).

Consideration should be given to the structure, chemical and mechanical properties of enamel in such severe cases if restoration is to be chosen. A systematic review of 22 studies of hypomineralised enamel identified an increase in porosity, a reduction in hardness and elasticity and a change in carbon–carbonate ratios when compared with normal enamel (Elhennawy et al. 2017). Additionally, an increase in protein content of serum albumin and other proteins has been associated with an inhibition of crystal growth and overall reduction in the mineral quantity and quality of MIH-affected enamel (Farah et al. 2010). As such, these characteristics should be considered when deciding how to restore these teeth.

*Restoration with composite resin* is a predictable option, with high success rates, if placed under rubber dam isolation to ensure good moisture control (Lygidakis et al. 2003; Kotsanos et al. 2005; Mejare et al. 2005; Rolim et al. 2021). The technique is simple, can be completed in one appointment and defective restorations can be easily repaired. Regarding the cavity design, total removal of hypomineralised enamel is suggested for the restoration to be successful (Lygidakis et al. 2010), as adhesion to remaining MIH-affected enamel is poorer with a reduced bond strength (Lagarde et al. 2020). Whilst there is a shift in caries management to minimally invasive approaches, poor success rates have been reported in the few studies that have placed composite restorations using a non-invasive approach for MIH-affected molars (Sonmez and Saat 2017; Linner et al. 2020). Pre-treatment with 5% sodium hypochlorite and the use of self-etch or total-etch techniques have been investigated clinically but the results thus far show that none of these techniques improves the success of the composite resin restorations substantially (Sonmez and Saat 2017; de Souza et al. 2017; Rolim et al. 2021; Somani et al. 2021). Overall, multiple studies report positive results but over a short follow-up period. Longer follow-up periods are necessary to substantiate this evidence. In addition, significant heterogeneity between studies existed as there were significant variations in the teeth studied including the size and extent of the defects, along with the presence or absence of atypical or typical carious lesions.

*Preformed metal crowns (PMC)* are an inexpensive option with reported high success rates (Kotsanos et al. 2005; Koleventi et al. 2018; Oh et al. 2020). They have the additional advantage of maintaining the structural integrity of the tooth whilst alleviating symptoms of hypersensitivity, maintaining the occlusal contact and can be placed in one visit making them ideal for use in teeth where multiple surfaces are involved. Prior to placement, clinical photography along with detailed clinical notes of the status of the tooth are essential, as once the crown is placed, it is impossible to assess the tooth without removing the crown and potentially causing further damage. Preparation of the tooth, with occlusal and proximal reduction, is usually required to achieve a good fit. The placement of orthodontic separators prior to the treatment appointment can be used to create the space required proximally, as this would diminish the need for interproximal tooth reduction. Nonetheless, it is still important to warn patients about a change in occlusion in the short-term. One study reported an increased periodontal pocket depth in the short-term (Koleventi et al. 2018). This may not be of clinical significance, especially if PMCs are used as an interim measure prior to scheduled extraction. However, further research is needed to understand the effects on the periodontium if they are to be used as a long-term option. The use of the Hall technique (Innes et al. 2007) for these teeth has also been suggested, however, further research is needed.

*Laboratory manufactured indirect restorations* have been studied more recently for MIH-affected molars. Like PMCs, these have reported very high success rates and can also be used in cases where multiple surfaces or cusps are involved and direct restorations would be inappropriate (Gaardmand et al. 2013; Dhareula et al. 2018, 2019; Linner et al. 2020). Three main categories of these restorations have been reported: metal alloys, indirect composite and ceramic restorations. All require technique sensitive tooth preparation, longer chair time and have an increased treatment cost. A temporary restoration may also be required in the interim period prior to final fit, which should be completed under rubber dam. They are generally placed supra-gingivally and therefore less likely to have an impact on the periodontium when compared to PMCs. Removal of all hypomineralised enamel is recommended to ensure appropriate bonding to clinically sound enamel. Metallic onlays are used for their superior wear resistance, strength, retention and durability, whilst materials such as gold can be placed in thin section (Harley and Ibbetson 1993; Zagdwon et al. 2003; Gaardmand et al. 2013; Dhareula et al. 2019;). Indirect composite onlays are a more aesthetic option and often require less preparation due to their adhesive properties (Dhareula et al. 2019). Furthermore, they can be easily repaired but do have a poorer wear resistance. Ceramic restorations are an aesthetic option with good strength and wear resistance noted but require greater tooth preparation (Linner et al. 2020).

Studies comparing the three types of indirect restorations, and PMCs, have found that all are successful, therefore, one technique or material cannot be recommended over another (Koch and Garcia-Godoy 2000; Zagdwon et al. 2003; Dhareula et al. 2019). Consideration needs to be given to use the least invasive option and extension of the restoration to conserve the remaining tooth structure due to wider pulp chambers, higher pulp horns and relatively shorter clinical crowns in children (Dhareula et al. 2019). Most studies had relatively small sample sizes and short follow-up periods, therefore, further longer-term studies with larger samples are needed to establish a definitive answer.

*Pulp therapy* in compromised first permanent molars is well documented, however, there is little evidence available specifically in MIH-affected molars. A recent systematic review on compromised first permanent molars found that partial and coronal pulpotomies have high success rates, in the short and long term, but there is limited evidence available for conventional pulpectomy or regenerative techniques (Taylor et al. 2020). Clearly this is an area where further research is needed, however, partial or coronal pulpotomies can be considered a potential treatment option in MIH-affected molars.

*Scheduled extractions* are indicated for teeth with significant breakdown, or for those that are pulpally involved or associated with a dental abscess or facial cellulitis. In severe cases, consideration should also be given to the long-term prognosis of the tooth, the likelihood of repeated dental interventions and the psychological impact on the child (Jälevik and Klingberg 2012). Extraction may be the best option in these cases but complete spontaneous space closure is not guaranteed, even if performed at the ideal time of 8–10 years of age (Ashley and Noar 2019). Three studies have reported variable success rates, in terms of spontaneous space closure, when MIH-affected molars are extracted (Mejare et al. 2005; Jälevik and Möller 2007; Oliver et al. 2014). These results are similar to those seen following extraction of MIH- and non-MIH-affected molars of poor prognosis (Eichenberger et al. 2015). This more radical approach appears also cost-effective compared to repeated restorative treatments, but there are no studies evaluating this parameter. To ensure the best possible outcome, orthodontic and radiographic evaluation is advised prior to scheduled extraction (Brusevold et al. 2021). An assessment of the child’s underlying malocclusion, any hypodontia, the presence or absence of crowding, the presence of the third permanent molar and the dental developmental stage of the child are required to aid the decision-making process (Ashley and Noar 2019).

b. **Treatment of anterior teeth**

Appropriate treatment decisions for anterior teeth must take into consideration several factors, as shown in Table 7.

**Table 7:** Factors to be considered for appropriate treatment planning of anterior teeth

At patient level	At oral level	At tooth level
Age of patient	Number of opacities	Colour of opacity
Medical history	DMFT	Size of opacity
Ability to cooperate	Developmental stage	Depth of opacity
Psychological impact of dental appearance on patient (e.g. bullying at school)		Presence/absence of sensitivity
Access to specialist dental care		Presence/absence of post-eruptive breakdown

*General considerations* Discoloured anterior teeth can have a considerable psychosocial impact on children (Rodd et al. 2011). Following treatment, an improvement in children’s overall health and oral health-related quality of life has been shown (Hasmun et al. 2020). A conservative approach is important in children due to the large pulp chambers, high pulp horns, and immature gingivae. Furthermore, a minimally invasive approach allows conservation of tooth structure for future restorative options. For children with poor oral hygiene, cariogenic diets and multiple carious teeth, cosmetic treatment should be deferred until an improvement is demonstrated and carious teeth treated. There are very few studies that are focussed on MIH-affected incisors with variable success rates reported. Consequently, recommendations for a particular approach cannot be made. Additionally, due to the variability of opacities and discolouration, a combination of techniques may be necessary. The use of rubber dam isolation, clinical photography before and after treatment in addition to an explanation of the limitations of treatment, is necessary for all options.

*Microabrasion* with either 18% hydrochloric acid or 37% phosphoric acid followed by casein phosphopeptide-amorphous calcium phosphate (CPP‑ACP) remineralizing agent appears to be effective for improving the aesthetic appearance of whitish creamy opacities (Bhandari et al. 2019). A pumice slurry or silicon carbide abrasive paste may be used. It is a minimally invasive approach, only removing when used appropriately, the surface 100–200 μm of enamel. As such, it is not suitable for deeper opacities (Wong and Winter 2002).

*Resin infiltration*, with a 15–20% hydrochloric acid etchant, ethanol and TEGDMA monomer infiltrant has been suggested for all types of opacities (Kim et al. 2011; ElBaz and Mahfouz 2017; Bhandari et al. 2018). It is a minimally invasive option and simple technique, that aims to improve the translucency, optical properties and overall colour of affected incisors (Crombie et al. 2014). Enhanced oral hygiene practices are essential as infiltrated enamel is more susceptible to staining (Ceci et al. 2017). The technique seems to be a feasible option for colour masking of whitish opacites in MIH, although there is no strong evidence supporting this (Borges et al. 2017). Additionally, the changes to enamel microhardness produced are still unpredictable (Crombie et al. 2014; Kumar et al. 2017) as resin infiltration depth is inconsistent and variable depending partly on pre-treatment protocols (Natarajan et al. 2015). However, since these lesions are usually located in non-stress surfaces, the alteration in microhardness should not affect the longevity of the treatment.

*Other non-invasive/micro-invasive treatment options* Whilst there are no studies specifically on MIH-affected incisors, previous research on external bleaching and the etch-bleach-seal technique for anterior opacities suggests that these may also be viable treatment options.

*The etch-bleach-seal technique* is a minimally invasive technique that can be used to remove yellow–brown stains (Wright 2002; Prud'homme et al. 2017), although its effectiveness has been questioned in MIH (Gandhi et al. 2012). The tooth is bleached with 5% sodium hypochlorite for up to twenty minutes, followed by application of 37% phosphoric acid etchant and clear resin sealant.

*External bleaching* is another non-invasive option that can be used in adolescents to camouflage white opacities by increasing the overall whiteness of the teeth. It is available as hydrogen peroxide (up to 6%) or carbamide peroxide (10% or 16%) gels used in custom-made trays. Its use by clinicians in Europe varies (Monteiro et al. 2020). Side effects include gingival irritation and sensitivity, and these should be considered seriously particularly when used in younger children. Currently, the EU (Directive 2011/84/EU of the European Commission, October, 29th 2011) restricts tooth whitening agents to 0.1% hydrogen peroxide in children, a clinically ineffective concentration (Griffiths and Parekh 2021).

*Composite restorations*, with or without the removal of enamel can mask opacities of all colours and replace areas where breakdown has occurred (Welbury 1991). Deeper opacities may require removal of enamel, but this should be performed as conservatively as possible due to the pulp anatomy of immature incisors. Thus, an opaquer may be required in specific cases, prior to composite being placed, to mask any yellow–brown discolouration without extensive enamel removal. Over time, marginal staining, wear, and fracture can occur and long-term maintenance of composite restorations is required (Welbury 1991; Wray and Welbury 2008).

*A combination of treatment approaches* may be the ideal future solution for MIH-affected anterior teeth. A recent study used a combination of approaches (microabrasion, resin infiltration, external bleaching and composite restoration) with each participant having an individualised approach depending on their clinical need; the results revealed that simple, minimally invasive treatments can provide good clinical and psychosocial outcomes (Hasmun et al. 2020). In addition, there are some case reports demonstrating these combined approaches (Attal et al. 2014; Prud'homme et al. 2017), however, further research is needed to investigate the efficacy and long-term outcomes of undertaking such combined treatment options in MIH-affected anterior teeth.

c. **Management of hypersensitivity and remineralisation**

Hypersensitivity can impact mastication, oral hygiene practices and quality of life. Several options for management are available and include casein phosphopeptide-amorphous calcium phosphate (CPP-ACP), casein phosphopeptide-amorphous calcium fluoride phosphate (CPP-ACFP), sodium fluoride varnish 5–6% with and without tricalcium phosphate, 8% arginine and calcium carbonate paste, ozone or low-level laser therapy. A recent systematic review reported that all studies which looked at the management of hypersensitivity showed a reduction post treatment, but none of these interventions can be recommended due to the moderate–high risk of bias in the studies, short follow-up times and small sample sizes (Somani et al. 2021). Similarly, the studies investigating remineralisation in MIH-affected teeth had comparable limitations (Somani et al. 2021). Remineralisation is difficult to measure, with an increase in laser fluorescence or quantitative light fluorescence readings reported in studies often not translating to a clinical improvement. However, despite these measurement challenges, topical CPP-ACP does seem to improve mineralisation due to the stabilisation of calcium and phosphate ions by casein phosphopeptide, a protein which carries these ions in the form of amorphous calcium phosphate (Baroni and Marchionni 2011; Biondi et al. 2017; Bakkal et al. 2017). As casein is a milk protein derivative, CPP-ACP should be avoided in those allergic to milk proteins. Although CPP-ACFP and sodium fluoride with and without tricalcium phosphate have shown to remineralise affected enamel (Restrepo et al. 2016; Bakkal et al. 2017; Biondi et al. 2017), there is insufficient evidence to recommend it for this purpose. Even though insufficient evidence exists for the use of fluoride varnish in remineralisation and desensitisation, it should still be used in children with MIH for caries prevention due to their increased risk.

*In general, *and regarding treatment of MIH-affected teeth, there is insufficient evidence to clearly suggest that any one of the above-detailed options is superior in the long term. Additionally, the majority of studies were rated at moderate or high risk of bias with significant heterogeneity preventing meta-analysis of the results in a recent systematic review (Somani et al. 2021).

### GRADE rating for the quality of evidence and strength of recommendations of the treatment studies in MIH teeth

Using the GRADE system, the rating for quality of evidence and strength of recommendation regarding treatment options for MIH molars and incisors in addition to remineralisation and sensitivity reduction options for MIH-affected teeth are shown in Tables 8, 9 and 10, respectively. Details of the implementation of all criteria regarding the included studies are shown in the supplement as Appendix 2.

**Table 8:** GRADE rating for quality of evidence and strength of recommendation regarding treatment options for MIH molars

Interventions for molars	No. of studies	No. of restorations /teeth^a^	GRADE of evidence quality	Strength of Recommendation
Fissure sealants, applied with an adhesive, can be used in mild cases in fully erupted molars	3	184	Moderate	Strong
GIC restorations using a non-invasive^b^ approach may be used as in cases where the child cannot co-operate for conventional treatment	5	333	Moderate	Conditional
Composite resin restorations placed under rubber dam isolation, using an invasive^ b^ approach can be used as a restorative option in mild/severe cases	8	793	Moderate	Strong
Non-invasive^ b^ composite restorations should not be placed	2	189	Moderate	Strong
The use of self-etch, total etch or deproteinisation with sodium hypochlorite is unlikely to make a difference to the retention rate of a composite restoration	3	137	Moderate	Strong
PMCs can be placed in severe cases	3	88	Moderate	Strong
Laboratory manufactured restorations using an invasive approach can be used as a restorative option in severe cases	4	132	Moderate	Conditional
Good space closure can be achieved spontaneously following extraction of affected molars	3	189	Moderate	Conditional

^a^Drop-outs have not been excluded as it was not possible to ascertain the number in all of the studies due to mixed data

^b^Non-invasive—preservation of affected enamel; invasive—removal of all hypomineralised enamel to achieve margin on clinically sound enamel

**Table 9:** GRADE rating for quality of evidence and strength of recommendation regarding treatment options for MIH incisors

Interventions for Incisors	No. of studies	No. of teeth	GRADE of evidence quality	Strength of Recommendation
Resin infiltration can be used to improve the appearance of affected incisor teeth	3	66	Low	Conditional
Microabrasion can be used to improve the appearance of affected incisor teeth	1	43	Very low	Conditional

**Table 10:** GRADE rating for quality of evidence and strength of recommendation regarding remineralisation and sensitivity reduction options for MIH-affected teeth

Interventions for remineralisation	No. of studies	No. of teeth	GRADE of evidence quality	Strength of Recommendation
Topical CPP-ACP can be used to remineralise affected teeth	3	61	Moderate	Conditional
Topical CPP-ACFP/NaF 4–5% with and without tricalcium phosphate can be used to remineralise affected teeth	3	88	Very low	Conditional

Interventions for reduction of hypersensitivity				
Topical CPP-ACP/CPP-ACFP/NaF 5–6% with and without tricalcium phosphate/8% arginine and calcium carbonate/ozone/laser can be used to reduce the symptoms of hypersensitivity in affected teeth	4	382	Low	Conditional

## Clinical practice guidance for treatment approach for MIH teeth

**Fig. 4:**
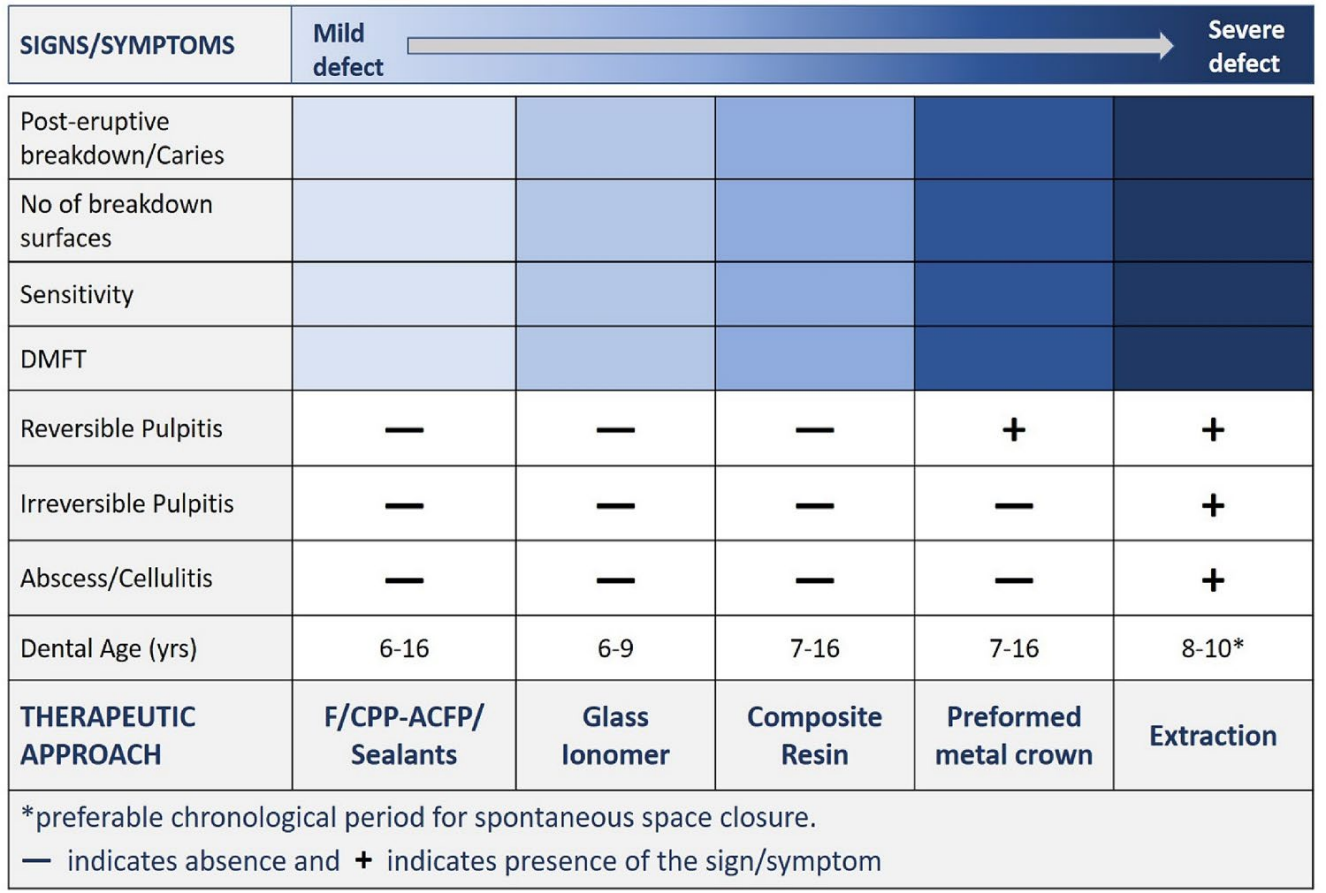
Diagrammatic management summary according to factor-severity for MIH-affected molars. Severity is incrementally increased from the left to the right. To select the appropriate option, the block with the most severe sign/symptom should dominate the choice

**Fig. 5:**
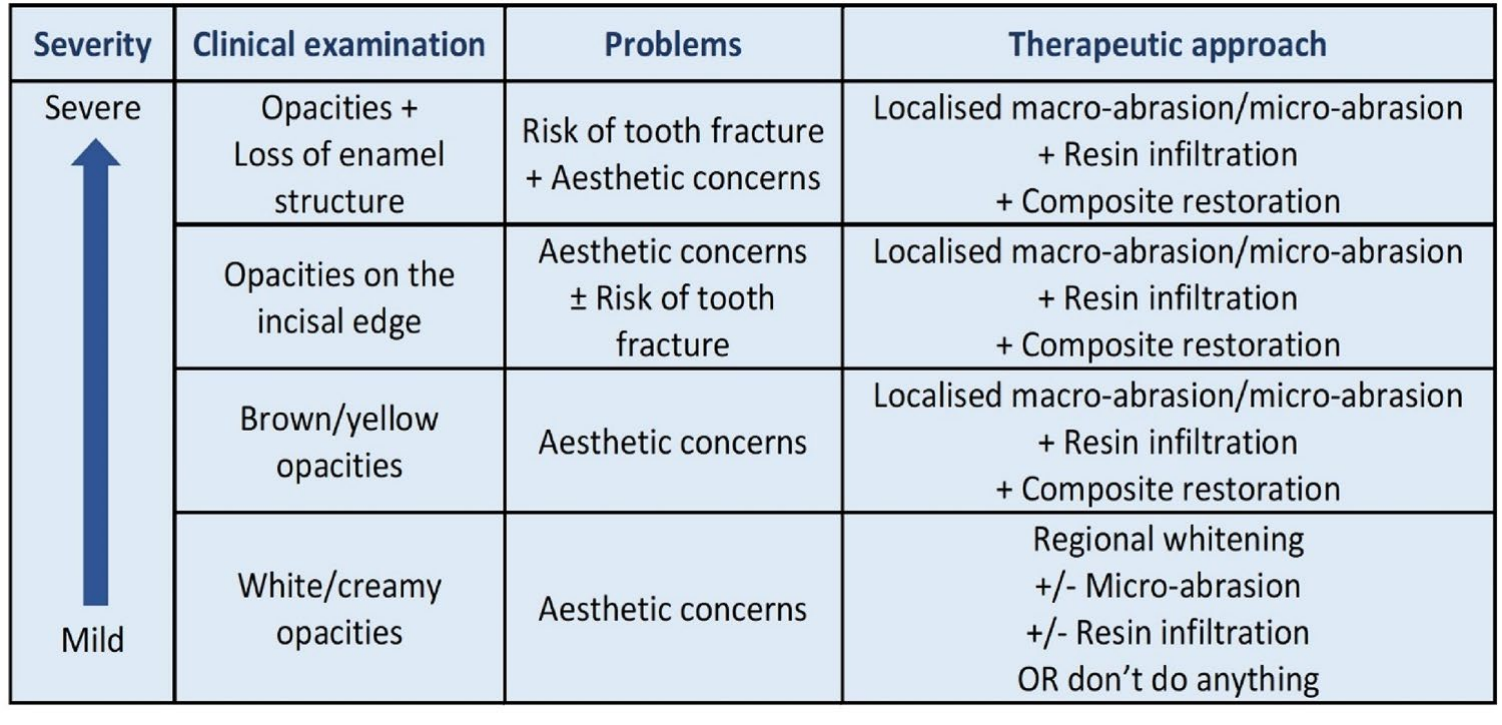
Diagrammatic management summary according to factor-severity for MIH-affected incisors. To select the appropriate option, the block with the most severe sign/symptom should dominate the choice

As this Policy Document was prepared to facilitate the clinician’s decision-making, a diagrammatic summary of possible factors interacting for each treatment modality according to the severity of the condition at a particular dental age, is shown in Figs. 4 and 5. They were constructed using sign/symptom-severity blocks to help the clinician to choose the appropriate treatment options.

## Conclusion 

- MIH is becoming an important public health issue with global prevalence of 12.9%–14.2% being reported. However, these figures may be an underestimation.
- The globally established EAPD diagnostic criteria and the recent efforts for unified and convenient charts for epidemiological studies will further help such studies in the future.
- The aetiology of MIH is better understood, as it clearly follows a multi-factorial model that in some instances may be the result of environmental–gene interactions. Systemic medical factors, such as perinatal hypoxia, prematurity and other hypoxia-related perinatal problems, including caesarean section appear to multiply the risk of having MIH. Infant and childhood illnesses, are also linked with MIH, while fever and antibiotic use, which may be considered as consequences to illnesses have been also been implicated.
  The role of genetic predisposition and epigenetic influences is becoming clearer, and may be regarded as the key piece of information currently missing to truly understand MIH aetiology.
- Successful preventive and treatment options have been studied and identified for MIH-affected molars, with the severity of the defect and the age of the patient often dictating the chosen approach. The same cannot be said for anterior teeth. Despite an increase in the number of studies addressing the management of MIH-affected teeth, the evidence is still limited with conventional restorative options remaining the most common approach.
- The EAPD strongly endorses the use of all available treatment tools for the treatment of MIH teeth, keeping in mind the necessity for painless and effective treatment plan and the well-being of the paediatric patient at dental, oral, medical and social levels.

## Future research recommendations

- Regarding the aetiology of MIH, future clinical studies should be prospective if systemic aetiological factors are to be evaluated, whilst genetic studies should focus on the genes and gene–environment interactions that regulate the genetic predisposition to MIH.
- Regarding the treatment of MIH, future research should focus on further improvements in adhesion, the use of new materials and the assessment of novel more minimally invasive techniques. Furthermore, to address the psychosocial and economic impacts of MIH treatments, a holistic management strategy should be adopted.

## Supplementary Information

Below is the link to the electronic supplementary material.

Supplementary file1 (DOCX 44 kb)

## Data Availability

Please contact corresponding author.
