# Supplementary material for: Best clinical practice guidance for clinicians dealing with children presenting with molar-incisor-hypomineralisation (MIH): an updated European Academy of Paediatric Dentistry policy document
Source: Eur Arch Paediatr Dent. 2021 Oct 20;23(1):3–21. doi: 10.1007/s40368-021-00668-5 (PMC8926988; doi:10.1007/s40368-021-00668-5)
Supplement: Supplementary file 1 — Supplementary file1 (DOCX 44 kb) [file 40368_2021_668_MOESM1_ESM.docx]

**APPENDIX 1**

**GRADE Criteria for MIH aetiology studies**

**DESIGN STUDY**: observational studies start with LOW quality of evidence

**RISK OF BIAS** Q1: how do we choose when the inclusion criteria of participants differ according to the studies included?

None of the studies are carried out completely blindly (-1)

| Study limitations in observational studies | |
| --- | --- |
|  | Explanation |
| Failure to develop and apply appropriate eligibility criteria (inclusion of control population) | - Under- or over-matching in case-control studies - Selection of exposed and unexposed in cohort studies from different populations |
| Flawed measurement of both exposure and outcome | - Differences in measurement of exposure (e.g. recall bias in case-control studies) - Differential surveillance for outcome in exposed and unexposed in cohort studies |
| Failure to adequately control confounding | - Failure of accurate measurement of all known prognostic factors - Failure to match for prognostic factors and/or adjustment in statistical analysis |
| Incomplete or inadequately short follow-up | Especially within prospective cohort studies, both groups should be followed for the same amount of time. |

**INDIRECTNESS**: e.g., breastfeeding for dioxins (-1) factor not directly measured

**INCONSISTENCY**: cf (compare/examine by comparison) heterogeneity results >50% (-1)

**IMPRECISION**: if confidence interval of OR is large (-1) and/or small sample (<500) (-1)

**PUBLICATION BIAS**: there is at least one factor for which the results are statistically positive so the other non-statistically significant results are also published + cf funnel plot

**LARGE MAGNITUDE OF EFFECT**: cf OR convert in RR and IC. If high (+1 or +2)

| Large | RR* >2 or <0.5  (based on direct evidence, with no plausible confounders) | May increase 1 level |
| --- | --- | --- |
| Very large | RR* >5 or <0.2  (based on direct evidence with no serious problems with risk of bias or precision, i.e., with (sufficiently narrow confidence intervals) | May increase 2 levels |

**DOSE-REPONSE**: duration (+1) e.g., breastfeeding or several antibiotics or duration of maternal smoking (but data is unknown for each study)

**CONFOUNDING FACTOR**: disease? treatment? fever? Indirect causes. (+1)

Confounding factor should increase the effect so if the effect and MIH are not significatively linked +1

Incubator / hypoxia

Asthma / asthma drug

Maternal disease / maternal medication

|  | **MIH aetiology studies. GRADE quality of evidence detailed.** | | | | | | | | | |
| --- | --- | --- | --- | --- | --- | --- | --- | --- | --- | --- |
| **Factors** | **study design** | **risk of bias** | **indirectness** | **inconsistency** | **imprecision** | **publication bias** | **large magnitude of effect** | **dose response** | **confounding factors** | **Total** |
| **Allergies** | LOW | 0 | 0 | 0 | 0 | 0 | 0 | 0 | 0 | **LOW** |
| **Antibiotics** | LOW | 0 | 0 | 0 | 0 | 0 | 0 | NA | 0 | **LOW** |
| **Asthma** | LOW | 0 | 0 | 0 | 0 | 0 | 0 | 0 | 0 | **LOW** |
| **Asthma drug** | LOW | 0 | -1 | 0 | 0 | 0 | 0 | NA | +1 | **LOW** |
| **Breast feeding >12months** | LOW | 0 | -1 | 0 | 0 | 0 | 0 | +1 | 0 | **LOW** |
| **Breast feeding >6months** | LOW | 0 | -1 | 0 | 0 | 0 | 0 | +1 | 0 | **LOW** |
| **Bronchitis** | LOW | 0 | 0 | 0 | 0 | 0 | 0 | NA | 0 | **LOW** |
| **Caesarean** | LOW | 0 | 0 | 0 | 0 | 0 | 0 | 0 | 0 | **LOW** |
| **Celiac disease** | LOW | 0 | 0 | 0 | -1 | 0 | 0 | 0 | 0 | **VERY LOW** |
| **Chicken pox** | LOW | 0 | 0 | -1 | 0 | 0 | 0 | 0 | 0 | **VERY LOW** |
| **Diarrhoea** | LOW | 0 | 0 | 0 | 0 | 0 | 0 | NA | 0 | **LOW** |
| **Eclampsia** | LOW | 0 | 0 | 0 | 0 | 0 | 0 | 0 | 0 | **LOW** |
| **Epigenetic (monozygotic twins)** | HIGH | 0 | 0 | 0 | 0 | 0 | +1 | 0 | 0 | **HIGH*** |
| **Fever** | LOW | 0 | 0 | 0 | 0 | 0 | 0 | NA | 0 | **LOW** |
| **Fluoride** | LOW | 0 | -1 | 0 | 0 | 0 | +1 | 0 | 0 | **LOW** |
| **Gastric disorders** | LOW | 0 | 0 | 0 | 0 | 0 | 0 | NA | 0 | **LOW** |
| **genetic (SNP association)** | HIGH | 0 | 0 | 0 | 0 | 0 | +1 | 0 | +1 | **HIGH*** |
| **Gestational diabetes** | LOW | 0 | 0 | 0 | -1 | 0 | 0 | NA | 0 | **VERY LOW** |
| **Gestational hypertension** | LOW | 0 | 0 | 0 | 0 | 0 | 0 | 0 | 0 | **LOW** |
| **Hypoxia at birth** | LOW | 0 | 0 | 0 | 0 | 0 | 0 | 0 | 0 | **LOW** |
| **Incubator** | LOW | 0 | 0 | 0 | 0 | 0 | 0 | 0 | +1 | **MODERATE** |
| **Jaundice** | LOW | 0 | 0 | 0 | -1 | 0 | 0 | NA | 0 | **VERY LOW** |
| **Kidney diseases** | LOW | 0 | 0 | 0 | -1 | 0 | +1 | NA | 0 | **LOW** |
| **Low weight at birth** | LOW | 0 | 0 | 0 | 0 | 0 | 0 | 0 | 0 | **LOW** |
| **Malnutrition** | LOW | 0 | 0 | 0 | -1 | 0 | 0 | NA | 0 | **VERY LOW** |
| **Maternal diseases** | LOW | 0 | 0 | 0 | 0 | 0 | 0 | NA | 0 | **LOW** |
| **Maternal fever** | LOW | 0 | 0 | 0 | 0 | 0 | 0 | NA | 0 | **LOW** |
| **Maternal smoking** | LOW | 0 | 0 | 0 | 0 | 0 | 0 | NA | 0 | **LOW** |
| **Maternal Urinary disease** | LOW | 0 | 0 | 0 | 0 | 0 | 0 | NA | 0 | **LOW** |
| **Measles** | LOW | 0 | 0 | 0 | 0 | 0 | 0 | NA | 0 | **LOW** |
| **Medication during pregnancy** | LOW | 0 | 0 | 0 | 0 | 0 | 0 | NA | +1 | **MODERATE** |
| **Otitis** | LOW | 0 | 0 | 0 | 0 | 0 | 0 | NA | 0 | **LOW** |
| **Pneumonia** | LOW | 0 | 0 | 0 | 0 | 0 | 0 | 0 | 0 | **LOW** |
| **Pre-eclampsia** | LOW | 0 | 0 | 0 | 0 | 0 | 0 | 0 | 0 | **LOW** |
| **Prematurity** | LOW | 0 | 0 | 0 | 0 | 0 | 0 | 0 | 0 | **LOW** |
| **Rhinitis** | LOW | 0 | 0 | 0 | 0 | 0 | 0 | NA | 0 | **LOW** |
| **Rubeola** | LOW | 0 | 0 | 0 | 0 | 0 | 0 | NA | 0 | **LOW** |
| **Sinusitis** | LOW | 0 | 0 | 0 | 0 | 0 | 0 | NA | 0 | **LOW** |
| **Throat infections** | LOW | 0 | 0 | 0 | 0 | 0 | 0 | NA | 0 | **LOW** |
| **Tonsillitis** | LOW | 0 | 0 | -1 | -1 | 0 | 0 | 0 | 0 | **VERY LOW** |
| **Urinary tract infection** | LOW | 0 | 0 | 0 | 0 | 0 | 0 | NA | 0 | **LOW** |
| **Vitamin D deficiency** | HIGH | 0 | 0 | 0 | 0 | 0 | 0 | +1 | +1 | **HIGH*** |
| **COMMENTS** | * All 3 studies rated as HIGH, are RCTs having a study design considered as High and the risk of bias in the measurement of each factor is low as it comes from either blood or oral samples (+1). Moreover, there is a large magnitude effect (RR>2) (+1). We could reduce the rate of study design to Moderate but the final result will still be High.  Concerning the 2 genetic studies, several SNP (single nucleotide polymorphisms) were searched, so we could record for each SNP the number of MIH patients, but we were afraid that this would make the table more cumbersome.  Concerning the 1 Vitamin D study, the parameter measured was not presence or absence but a continuous variable (Serum 25(OH)D concentration). | | | | | | | | | |

**APPENDIX 2**

**GRADE Criteria for MIH treatment studies**

Studies looking at an individual treatment option were grouped together before and assessment was made. Any management option that had at least one randomised trial was initially rated as high. Any management option that had no randomised trials (i.e., observational studies only) was rated initially as low. The assessment varies for these studies and those starting at a low grading, were only downgraded further to very low if there was serious and significant uncertainty with the evidence.

Assessment was based on the eighth GRADE criteria and the rationale for assessment is outlined below. Due to high heterogeneity in the studies, a meta-analysis was not performed making assessment difficult. Factors such as confidence intervals, the I^2^ and p-values have therefore not been included in the below criteria.

**Risk of Bias**

- Based on the risk of bias assessment in the systematic review where an overall rating was given to each study
- For randomised trials the RoB 2 tool was used
- For non-randomised trials the ROBINS-I tool was used
- The studies were grouped and the highest risk of bias rating was used
- Where it was high/serious, the initial rating was downgraded by 1

**Imprecision**

The certainty was downgraded by one if:

- If there were ≤2 studies
- Small sample size

**Inconsistency:**

The certainty was downgraded by one if:

- High heterogeneity in results (e.g. difference of >50% in success rates, contradictory results)

**Indirectness**

The certainty was downgraded by one if:

- Very different and incomparable outcome measures were used
- Inappropriate outcome measures were used
- Multiple sub-groups were present leading to indirect comparisons

**Publication bias**

The certainty was downgraded by one if:

- A study was biased in their reporting of the results and outcomes
- Industry sponsored studies

**Large magnitude of effect**

The certainty was upgraded by one if:

- A high success rate was reported in at least 3 studies

**Dose-response gradient**

The certainty was upgraded by one if:

- There was evidence of a dose-response gradient i.e., an increase in the concentration of fluoride varnish placed would have led to a increased reduction of hypersensitivity

**Confounding factors**

The certainty was upgraded by one if:

- All plausible confounders would have reduced the effect

**References:**

- GRADE: https://www.gradeworkinggroup.org/
- Cochrane Handbook: https://training.cochrane.org/handbook/current/chapter-14
- BMJ Best Practice Toolkit: <https://bestpractice.bmj.com/info/toolkit/learn-ebm/what-is-grade/>

**GRADE quality of evidence for included studies for treatment of MIH.**

| **Intervention** | **No studies** | **Mean age participants* (years)** | **Mean follow-up* (months)** | **GRADE QUALITY OF EVIDENCE** |
| --- | --- | --- | --- | --- |
| *Molars*  **Fissure sealant** | 3 | 7.16 | 40 | MODERATE |
| **Amalgam** | 2 | 8.10 | 54 | VERY LOW |
| **Compomer** | 1 | 8.50 | NR | VERY LOW |
| **Composite** | 8 | 8.67 | 31.84 | MODERATE |
| **GIC** | 5 | 9.38 | 22.70 | MODERATE |
| **Lab-made restorations** | 4 | 11.2 | 38.05 | MODERATE |
| **PMC** | 3 | 9.19 | 34.76 | MODERATE |
| **Extraction (space closure)** | 3 | 8.93 | 56.40 | MODERATE |
| *Incisors* |  |  |  |  |
| **Microabrasion** | 1 | NR | 6.00 | VERY LOW |
| **Resin infiltration** | 3 | NR | 2.42 | LOW |
| *Hypersensitivity* |  |  |  |  |
| **Arginine & calcium carbonate (8%)** | 1 | NR | 2.00 | VERY LOW |
| **CPP-ACP** | 2 | NR | 3.00 | LOW |
| **CPP-ACFP** | 1 | NR | 3.00 | LOW |
| **Laser** | 1 | NR | 1.00 | LOW |
| **NaF (5-6%)** | 2 | NR | 2.00 | LOW |
| **Ozone** | 1 | NR | 3.00 | LOW |
| *Remineralisation* |  |  |  |  |
| **CPP-ACP** | 3 | NR | 12.83 | MODERATE |
| **CPP-ACFP** | 1 | NR | 1.00 | VERY LOW |
| **NaF (4-5%)** | 2 | NR | 1.25 | VERY LOW |
| **NaF containing tricalcium phosphate (TCP)** | 1 | NR | 1.50 | VERY LOW |
| **COMMENTS** | **These figures are approximations and taken from the available data; where data were not available or mixed, they were excluded from the mean calculation.* | | | |

|  | **MIH treatment studies. GRADE quality of evidence detailed.** | | | | | | | | | |
| --- | --- | --- | --- | --- | --- | --- | --- | --- | --- | --- |
| **Intervention** | **Study design** | **Risk of bias** | **Indirectness** | **Inconsistency** | **Imprecision** | **Publication bias** | **Large magnitude of effect** | **Dose response** | **Confounding factors** | **Final** |
| *Molars*  **Fissure sealant** | **HIGH** | -1 | 0 | 0 | 0 | 0 | 0 | NA | 0 | MODERATE |
| **Amalgam** | **LOW** | 0 | -1 | 0 | -1 | 0 | 0 | NA | 0 | VERY LOW |
| **Compomer** | **LOW** | 0 | NA | NA | -1 | 0 | 0 | NA | 0 | VERY LOW |
| **Composite** | **HIGH** | -1 | -1 | 0 | 0 | 0 | +1 | NA | 0 | MODERATE |
| **GIC** | **LOW** | 0 | 0 | 0 | 0 | 0 | +1 | NA | 0 | MODERATE |
| **Lab-made restoration** | **HIGH** | -1 | -1 | 0 | 0 | 0 | +1 | NA | 0 | MODERATE |
| **PMC** | **LOW** | 0 | 0 | 0 | 0 | 0 | +1 | NA | 0 | MODERATE |
| **Extraction (space closure)** | **LOW** | 0 | 0 | 0 | 0 | 0 | +1 | NA | 0 | MODERATE |
| *Incisors* |  |  |  |  |  |  |  |  |  |  |
| **Microabrasion** | **HIGH** | -1 | NA | NA | -2 | 0 | 0 | NA | 0 | VERY LOW |
| **Resin infiltration** | **LOW** | 0 | 0 | 0 | 0 | 0 | 0 | NA | 0 | LOW |
| *Hypersensitivity* |  |  |  |  |  |  |  |  |  |  |
| **Arginine & calcium carbonate (8%)** | **LOW** | 0 | 0 | NA | -1 | -1 | 0 | NA | 0 | VERY LOW |
| **CPP-ACP** | **HIGH** | -1 | 0 | 0 | -1 | 0 | 0 | NA | 0 | LOW |
| **CPP-ACFP** | **HIGH** | -1 | 0 | NA | -1 | 0 | 0 | NA | 0 | LOW |
| **Laser** | **HIGH** | -1 | 0 | NA | -1 | 0 | 0 | NA | 0 | LOW |
| **NaF (5-6%)** | **HIGH** | -1 | 0 | 0 | -1 | 0 | 0 | No | 0 | LOW |
| **Ozone** | **HIGH** | -1 | 0 | NA | -1 | 0 | 0 | NA | 0 | LOW |
| *Remineralisation* |  |  |  |  |  |  |  |  |  |  |
| **CPP-ACP** | **LOW** | 0 | 0 | 0 | 0 | 0 | +1 | NA | 0 | MODERATE |
| **CPP-ACFP** | **LOW** | 0 | 0 | 0 | -2 | 0 | 0 | NA | 0 | VERY LOW |
| **NaF (4-5%)** | **HIGH** | -1 | 0 | -1 | -1 | 0 | 0 | No | 0 | VERY LOW |
| **NaF containing tricalcium phosphate (TCP)** | **LOW** | 0 | 0 | 0 | -2 | 0 | 0 | NA | 0 | VERY LOW |
| **COMMENTS** | The GRADE quality of evidence assessment was made for each of the different treatment options based on the best available evidence which was included in the systematic review (Somani et al. 2021).  The GRADE strength of the recommendations was based on the GRADE quality assessment, other evidence that was valuable, however, was not included in the systematic review as it did not meet the criteria (i.e., in vitro studies, case series) and finally based on consensus from all of the authors based on their clinical experience and knowledge. This was necessary, as overall, there was a lack of high-quality research across all of the management strategies available. | | | | | | | | | |
